# The role of interleukin-33 in patients with mild cognitive impairment and Alzheimer’s disease

**DOI:** 10.1186/s13195-020-00652-z

**Published:** 2020-07-16

**Authors:** Chih-Sung Liang, Kuan-Pin Su, Chia-Lin Tsai, Jiunn-Tay Lee, Che-Sheng Chu, Ta-Chuan Yeh, Ming-Wei Su, Guan-Yu Lin, Yu-Kai Lin, Hsuan-Te Chu, Chia-Kuang Tsai, Fu-Chi Yang

**Affiliations:** 1 Department of Psychiatry, Beitou Branch, Tri-Service General Hospital, National Defense Medical Center, Taipei, Taiwan; 2 Graduate Institute of Medical Sciences, National Defense Medical Center, Taipei, Taiwan; 3 Department of Psychiatry & Mind-Body Interface Laboratory (MBI-Lab), China Medical University Hospital, Taichung, Taiwan; 4 College of Medicine, China Medical University, Taichung, Taiwan; 5 An-Nan Hospital, China Medical University, Tainan, Taiwan; 6 Department of Neurology, Tri-Service General Hospital, National Defense Medical Center, No.325, Section 2, Cheng-Kung Road, Neihu District, Taipei City 114, Taiwan; 7 Department of Psychiatry, Kaohsiung Veterans General Hospital, Kaohsiung, Taiwan; 8 Center for Geriatric and Gerontology, Kaohsiung Veterans General Hospital, Kaohsiung, Taiwan; 9 Department of Psychiatry, Tri-Service General Hospital, National Defense Medical Center, Taipei, Taiwan; 10 Institute of Biomedical Sciences, Academia Sinica, Taipei, Taiwan

**Keywords:** Cytokine, Interleukin-33, Alzheimer’s disease, Mild cognitive disorder, Cognitive decline

## Abstract

**Background:**

The neuroprotective role of interleukin (IL)-33 is supported by numerous preclinical studies, but it remains uninvestigated in clinical studies of Alzheimer’s disease (AD). We aimed to examine the association between human blood levels of IL-33 and cognitive preservation in amnestic mild cognitive impairment (aMCI) and AD.

**Methods:**

A total of 100 participants (26 controls, 35 aMCI patients, and 39 AD patients) completed two Mini-Mental State Examinations (MMSEs) over a 1-year interval. In all 100 participants at the second MMSE, we examined the plasma levels of IL-33, IL-β, IL-1 receptor agonist (IL-1RA), beta amyloid (Aβ), and tau and apolipoprotein E (ApoE) genotyping; we also performed Hopkins Verbal Learning Test, Trail Making Test, forward and backward digit span, and Clinical Dementia Rating.

**Results:**

IL-33 expression showed a positive trend among controls (1/26 = 3.8%), aMCI (9/35 = 25.7%), and AD (17/39 = 43.6%) (trend analysis: *P* < 0.001). Patients expressing IL-33 preserved their cognitive function compared with IL-33 non-expressing patients (1-year ΔMMSE, 0.16 ± 1.6 vs − 1.5 ± 2.6; *P* = 0.006). The cognitive preservation was not associated with the lower levels of Aβ, tau, and ApoE ε4, while higher levels of ApoE ε4 and phosphorylated tau were indeed associated with cognitive decline. The aMCI patients with AD conversion during study period had higher proportion of IL-33(−) than non-AD converters (90.9% vs 53.3%, *P* = 0.04).

**Conclusions:**

IL-33 or its associated signaling pathways may represent a new treatment paradigm for aMCI and AD.

## Background

The main pathophysiology of Alzheimer’s disease (AD) involves the accumulation of insoluble forms of amyloid-beta (Aβ) peptide into plaques and the aggregation of the microtubule protein tau into neurofibrillary tangles [1–5]. In addition to the amyloid and tau hypotheses, substantial evidence suggests that innate immune system-mediated actions drive and exacerbate AD pathogenesis [6–10]. Importantly, in the preclinical stages of AD, neuroinflammation triggers a vicious cycle of microglial activation, release of pro-inflammatory factors, and neuronal damage. Exploration of innate immune-mediated mechanisms and the use of immunomodulation as a disease-modification strategy have been promising in the preclinical research of AD [11]. Animal studies have shown that targeting the innate immune molecules or their respective signaling pathways may substantially ameliorate AD-related pathology [11–14].

Interleukin (IL)-33 is a member of the IL-1 family and broadly expressed in stromal and barrier tissue, including oligodendrocytes and astrocytes in the central nervous system (CNS) [15, 16]. Although IL-33 is originally thought to be a cellular alarmin released from nuclear stores after tissue damage, new in vivo data found that astrocyte-derived IL-33 is the key molecule promoting synapse refinement by microglia during CNS development [17]. Increasing evidence also indicates the critical role of IL-33 in shaping type 1, type 2, and regulatory immune responses [15]. Supporting IL-33 as a therapeutic target in AD comes from animal and human cellular and genetic studies [18–20]. An animal study showed that peripheral IL-33 administration reduced soluble Aβ levels and amyloid plaque deposition and reversed synaptic plasticity impairment and cognitive decline in AD mouse models [18]. Another animal study reported that IL-33 deficiency caused tau abnormality, neurodegeneration, and AD-like symptoms in aged mice [19]. Consistent with these findings, a human genetic study showed that IL-33 expression is reduced in the brains of individuals with AD [21]. Another human study reported that when compared with the mild cognitive impairment (MCI) patients with subsequent AD conversion, the MCI patients without AD conversion had higher levels of IL-33^+^ cells that were also positively correlated with hippocampus volumes [20]. These findings suggested the potential therapeutic role of IL-33 in AD.

To date, no human study has examined the association between IL-33 and cognitive preservation in MCI and AD. Here, we showed that < 50% of MCI and AD patients had peripheral IL-33 expression. Also, the IL-33-expressing patients preserved their cognitive function over 1-year period compared with the patients without IL-33 expression. The cognitive preservation was not associated with the levels of Aβ and tau protein, the risk factors of AD. In contrast, higher apolipoprotein E (ApoE) ε4 expression and higher levels of phosphorylated tau 181 (p-Tau) were associated with rapid cognitive decline. Moreover, IL-33 non-expression was associated with AD conversion in the MCI patients. Collectively, this is the first human study supporting the association between the peripheral IL-33 expression and cognitive preservation in MCI and AD.

## Methods

### Subjects and study design

The protocol was approved by the Institutional Review Board for the Protection of Human Subjects at the Tri-Service General Hospital (TSGHIRB 1-107-05-111). A total of 109 participants aged between 64 and 88 years were recruited between January 2015 and December 2018 at the memory clinic at the Tri-Service General Hospital of the National Defense Medical Center, Taiwan. Individuals were eligible if they had negative findings on physical and neurological examinations, laboratory tests (creatinine, fasting blood sugar, free-thyroxine 4, high-sensitivity thyroid stimulating hormone, vitamin B12, folic acid, serologic test for syphilis, and routine blood tests), and neuroimaging examinations (brain computed tomography or magnetic resonance imaging).

Participants underwent a baseline Mini-Mental Status Examination (MMSE) at recruitment. After 1-year follow-up, the following cognitive tests were performed, including MMSE, Clinical Dementia Rating (CDR), short-form Geriatric Depression Scale (GDS-S), Hopkins Verbal Learning Test (HVLT), forward and backward digit span, Trail Making Test Part A (TMTA), and Hachinski Ischemia Scale (HIS).

Individuals were excluded if they had the following: (a) a history of major or uncontrolled medical condition, such as heart failure, sepsis, liver cirrhosis, renal failure, chronic obstructive pulmonary disease, and poorly controlled diabetes (hemoglobin A1c > 8.5), myocardial infarction, or malignancy; (b) substance abuse; (c) a history of major neurological disorders, such as stroke or Parkinson’s disease; (d) GDS-S > 9 or modified Rankin Scale scores > 3; and (e) a history of major psychiatric condition that can impair cognition, such as major depressive disorder, bipolar disorder, or schizophrenia.

Participants were allocated to the control group, MCI due to AD (aMCI) group, or AD group based on the results of HVLT, MMSE, CDR, and HIS and the recommendations from the National Institute on Aging-Alzheimer’s Association (NIA-AA) workgroups on diagnostic guidelines for AD and aMCI [22, 23]. Normal controls were required to satisfy the following: (a) no active neurological or psychiatric disorders; (b) no psychotropic drugs; (c) MMSE > 26 (middle school), MMSE > 22 (primary school), and MMSE > 19 (illiteracy); and (d) CDR score = 0. In addition to NIA-AA criteria [22], aMCI was required to satisfy the following criteria: (a) CDR = 0.5; (b) MMSE > 26 (middle school), MMSE > 22 (primary school), and MMSE > 19 (illiteracy); (c) HIS ≤ 3; and (d) HVLT ≤ 22 [24]. In addition to NIA-AA criteria [23], AD was required to satisfy the following criteria: (a) CDR ≥ 0.5; (b) MMSE ≤ 26 (middle school), MMSE ≤ 22 (primary school), and MMSE ≤ 19 (illiteracy); (c) HIS ≤ 3; and (d) HVLT ≤ 19 [24].

### Preparation of plasma samples

Fasting blood was drawn using 9-mL K3-EDTA tubes (455036, Greiner Bio-one GmbH, Kremsmünster, Austria), which were gently inverted three times immediately following blood collection. Blood samples were then centrifuged at a relative centrifugal force (2300*g*) for 10 min (4 °C) using a swing-out (bucket) rotor (5202R, Eppendorf, Hamburg, Germany). Each 0.4-mL plasma sample was transferred to a fresh 2.0-mL tube (CryzoTraq, Ziath, Cambridge, UK). All plasma samples were stored in 0.5 mL aliquots at − 80 °C within 8 h of blood collection. For the measurements of the cytokine levels, the plasma samples were thawed on ice, and 50-μL aliquots were prepared and stored at − 80 °C.

### Plasma levels of Aβ and tau protein

Immunomagnetic reduction (IMR), an ultra-sensitive analytical assay method, can reliably assay ultra-low concentrations of human blood biomarkers, including Aβ_1-40_, Aβ_1-42_, total tau (t-Tau), and p-Tau181 [25]. For each plasma sample, the levels of Aβ_1-40_, Aβ_1-42_, t-Tau, and p-Tau181 were assayed using IMR kits (MF-AB0-0060, MF-AB2-0060, MF-TAU-0060, and MF-PT1-0060, MagQu Co., New Taipei City, Taiwan). For each assay, 40 μL (Aβ_1-40_, t-Tau, and p-Tau181) or 60 μL (Aβ_1-42_) of plasma was mixed with 80 or 60 μL of reagent, respectively. Each reported biomarker concentration represents the average of duplicated measurements. An IMR analyzer (XacPro-S, MagQu Co., New Taipei City, Taiwan) was used for all assays. The reliability of IMR measurements ranged from 0.17 to 1000 pg/mL for Aβ_1-40_, 0.77 to 30,000 pg/mL for Aβ_1-42_, 0.026 to 3000 pg/mL for t-Tau, and 0.0196 to 1000 pg/mL for p-Tau181. The intra-assay or inter-assay coefficient of assay variation using IMR was within the range of 7 to 10% for high-concentration quality control samples of Aβ_1-40_, Aβ_1-42_, t-Tau, or p-Tau181. For low-concentration quality control samples of Aβ_1-40_, Aβ_1-42_, t-Tau, or p-Tau181 using IMR, the intra-assay or inter-assay coefficient of assay variation was within the range of 10 to 15%. For each kind of biomarker, two batches of reagent were used. The quality of each batch of reagents was well controlled by monitoring particle size, particle concentration, and bioactivity. The variation in these reagent properties between batches is lower than 10%.

### Plasma levels of cytokines

A multiplex bead array assay was used to examine plasma levels of cytokines. The detailed procedures for detection of soluble cytokines by multiplex bead array assays have been previously reported [26, 27]. Three cytokines (IL-1β, IL-1 receptor antagonist (RA), and IL-33) were determined by using a customized human cytokine magnetic bead panel (Bio-Rad; Yu-Shing Biotech., Ltd., Taipei, Taiwan) according to the manufacturer’s instructions (Bio-Rad; Genmall Biotechnology Co., Ltd., Taipei, Taiwan). The median fluorescence intensities were collected on a Bio-Plex 200 instrument (Bio-Rad) using Bio-Plex Manager software version 6.0 (Bio-Rad). Study samples were tested in duplicate, and the duplicate measurements were averaged for statistical analysis. Standard curves were created from duplicate values, and all samples were analyzed as single determinations. All analyses were performed in one batch using kits from the same production lot.

### ApoE genotyping

To efficiently obtain genetic information from samples collected from Taiwanese patients of Han Chinese ethnicity, the Taiwan Biobank (TWB) designed the TWB genotype array, based on the Affymetrix Axiom genotyping platform. The TWB genotype array enabled good-quality genotyping. Two single-nucleotide polymorphisms (SNPs, rs429358 and rs7412) defining ApoE isoforms were genotyped using the TWB array.

### Statistical analysis

Categorical variables were analyzed using Pearson’s chi-square test, and continuous variables were analyzed using Student’s *t* test or the Mann-Whitney test. Trend analysis was analyzed using the Cochran-Armitage test. Spearman’s rank-order correlation analysis was carried out to examine the association between MMSE score and IL-33 levels. All tests were two-sided, and *P* < 0.05 was considered significant. Error bars represent mean ± standard deviation. All statistical analyses were performed using SPSS software version 25.0 (IBM SPSS, IBM Corp., Armonk, NY, USA) and GraphPad Prism software version 8.0 (GraphPad Software, San Diego, CA, USA).

## Results

### The demographics and cognitive performance

A total of 100 individuals fulfilled the study criteria and completed the second MMSE, of which 26 were healthy controls (HC), 35 were aMCI, and 39 were AD. We first examined the group differences in demographics and cognitive performance (Table 1). The patient group (aMCI plus AD) was older (*P* < 0.001) and had higher education level (*P* = 0.02) than the control group. Their performance on all of the cognitive tests (all *P* < 0.001) was poorer than that of healthy controls.

**Table 1:** Clinical characteristics of participants

	Patient group vs control group			Patient group		
	Controls (***N*** = 26)	Patients (***N*** = 74)	***P***	aMCI (***N*** = 35)	AD (***N*** = 39)	***P***
Female (%)	21 (80.8%)	52 (70.3%)	0.30	23 (65.7%)	29 (74.4%)	0.42
Age (year)	68.7 ± 4.2	76.7 ± 8.5	**< 0.001**	75.6 ± 8.4	77.7 ± 8.5	0.30
Body mass index	23.7 ± 3.3	24.4 ± 3.4	0.38	24.5 ± 3.2	24.3 ± 3.7	0.82
Education (year)	11.4 ± 4.2	8.8 ± 4.9	**0.02**	9.2 ± 5.1	8.4 ± 4.7	0.51
First MMSE	28.4 ± 0.8	23.9 ± 5.8	**< 0.001**	26.9 ± 2.3	21.2 ± 6.7	**< 0.001**
24–30	26 (100.0%)	50 (67.6%)	**0.011**	32 (91.4%)	18 (46.2%)	**< 0.001**
19–23	0 (0.0%)	11 (14.9%)		3 (8.6%)	8 (20.5%)	
10–18	0 (0.0%)	12 (16.2%)		0 (0%)	12 (30.8%)	
0–9	0 (0.0%)	1 (1.4%)		0 (0%)	1 (2.6%)	
Second MMSE	28.4 ± 1.5	22.9 ± 5.8	**< 0.001**	26.5 ± 2.0	19.7 ± 6.2	**< 0.001**
Hopkins Verbal Learning Test	23.4 ± 3.7	15.0 ± 4.9	**< 0.001**	16.7 ± 4.4	13.5 ± 4.9	**0.005**
Forward digit span	11.7 ± 1.7	9.0 ± 2.6	**< 0.001**	9.3 ± 2.5	8.6 ± 2.6	0.26
Backward digit span	7.2 ± 2.7	3.8 ± 2.4	**< 0.001**	4.7 ± 2.3	3.0 ± 2.1	**0.001**
Trail Making Test Part A	56.7 ± 25.6	115.3 ± 86.5	**< 0.001**	87.6 ± 67.7	140.2 ± 94.5	**0.007**
Clinical Dementia Rating	0.04 ± 0.14	1.05 ± 0.72	**< 0.001**	0.50 ± 0.00	1.54 ± 0.68	**< 0.001**
Apolipoprotein E ε2:ε3:ε4	7:12:7 (27%:46%:27%)	9:50:15 (12%:68%:20%)	0.109	2:28:5 (6%:80%:14%)	7:22:10 (18%:56%:26%)	0.084

Data are presented as mean ± standard deviation or frequency (percentage)

*Abbreviation*: *aMCI* mild cognitive impairment due to Alzheimer’s disease, *AD* Alzheimer’s disease, *MMSE* Mini-Mental State Examination

We examined the demographic and cognitive differences between aMCI and AD patients. Compared with aMCI patients, AD patients showed poorer performance on the first (*P* < 0.001) and second MMSE (*P* < 0.001), HVLT (*P* = 0.005), backward digit span (*P* = 0.001), TMTA (*P* = 0.007), and CDR (*P* < 0.001). The groups did not differ in female proportion, age, body mass index, and education levels.

### The peripheral levels of IL-33 in HC, aMCI, and AD

**Fig. 1:**
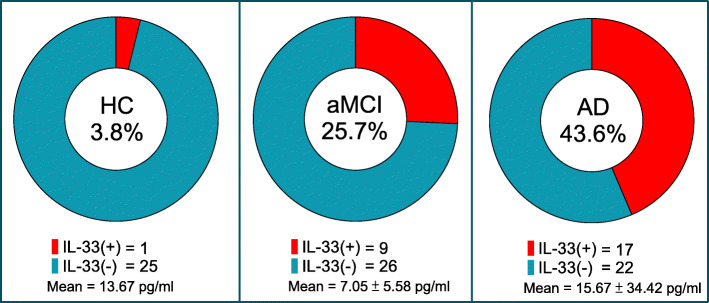
Patients with aMCI or AD had higher proportion of peripheral IL-33 expression with linear trend. Pearson’s Chi-Square test: *P* = 0.002; Cochran–Armitage test for linear trend analysis: *P* < 0.001. Abbreviations: *aMCI* amnestic mild cognitive impairment, *AD* Alzheimer’s disease, *HC* healthy controls, *IL* interleukin

We next sought to examine the peripheral levels of IL-33 among the three groups. The lowest limit of detection for IL-33 was 0.2 pg/mL. Among the 100 samples, the IL-33 levels were detectable only in 28 samples. We analyzed IL-33 data as a binary variable. IL-33(+) indicates detectable IL-33 levels and IL-33(−) undetectable. The proportion of IL-33(+) and the plasma concentration of IL-33 in each group are shown in Fig. 1. The three groups had significantly different proportion of IL-33(+) (*P* = 0.002), and HC, aMCI, and AD showed a positive linear trend (3.8%, 25.7%, 43.6%; *P* < 0.001).

### The peripheral levels of Aβ_1-42_, Aβ_1-40_, t-Tau, p-Tau, IL-1β, and IL-1RA

To explore group differences in AD-related biomarkers and cytokine levels, we examined the peripheral levels of Aβ_1-42_, Aβ_1-40_, t-Tau, p-Tau, IL-1β, and IL-1RA in the three groups (Table 2). As expected, the patient group had higher levels of Aβ_1-42_ (17.3 ± 1.0 vs 16.9 ± 0.7, *P* = 0.038), t-Tau (26.6 ± 5.1 vs 23.8 ± 3.9, *P* = 0.014), and p-Tau (4.1 ± 0.9 vs 3.7 ± 0.7, *P* = 0.035) than the control group. The levels of these biomarkers were higher in AD than in aMCI patients, but not statistically significant. The other two IL-1 families (IL-1β and IL-1RA) were 100% detectable in the control and the patient groups. The patient group had higher levels of IL-1β (0.12 ± 0.11 vs 0.07 ± 0.04, *P* = 0.039) and IL-1RA (96 ± 79 vs 63 ± 31, *P* = 0.045) compared with the control group. The comparisons between aMCI and AD in IL-1β and IL-1RA levels were not statistically significant.

**Table 2:** IMR data and cytokine levels

	Patient group vs control group			Patient group		
	Controls (***N*** = 26)	Patients (***N*** = 74)	***P***	aMCI (***N*** = 35)	AD (***N*** = 39)	***P***
Aβ_1-40_	50.7 ± 4.9	52.1 ± 4.2	0.160	51.9 ± 4.8	52.3 ± 3.7	0.654
Aβ_1-42_	16.9 ± 0.7	17.3 ± 1.0	**0.038**	17.2 ± 1.1	17.4 ± 0.9	0.455
t-Tau	23.8 ± 3.9	26.6 ± 5.1	**0.014**	26.0 ± 5.1	27.1 ± 5.2	0.374
p-Tau181	3.7 ± 0.7	4.1 ± 0.9	**0.035**	4.0 ± 0.9	4.2 ± 0.9	0.340
IL-1β	0.07 ± 0.04	0.12 ± 0.11	**0.039**	0.12 ± 0.11	0.12 ± 0.12	0.765
IL-1RA	63 ± 31	96 ± 79	**0.045**	108 ± 104	85 ± 45	0.289

*Abbreviation*: *aMCI* mild cognitive impairment due to Alzheimer’s disease, *AD* Alzheimer’s disease, *IL* interleukin; *IL-1RA* interleukin 1 receptor antagonist

### The association between IL-33 expression and cognitive preservation

To determine whether IL-33 expression might be a protective factor for aMCI and AD patients, we examined the association between IL-33 expression and 1-year change in MMSE. The aMCI and AD patients were divided into two groups: IL-33(+) and IL-33(−). The IL-33(+) patients did not differ from the IL-33(−) patients in female proportion (76.9% vs 66.7%, *P* = 0.357), age (77.0 ± 8.6 vs 76.6 ± 8.5, *P* = 0.848), education levels (8.4 ± 5.1 vs 9.0 ± 4.8, *P* = 0.632), and BMI (24.0 ± 3.5 vs 24.6 ± 3.4, *P* = 0.455).

**Fig. 2:**
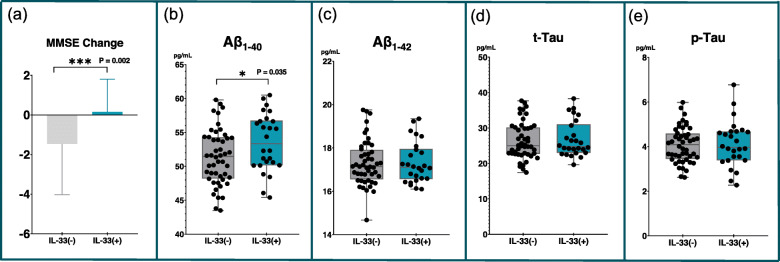
Patients with IL-33 expression may preserve cognitive function although having high levels of Aβ_1-40_. Error bars indicate standard deviation. Abbreviations: *Aβ* amyloid β, *MMSE* Mini Mental Status Examination, *p-Tau* phosphorylated Tau 181, *t-Tau* total Tau

Figure 2 shows that the IL-33(+) patients significantly preserved their general cognitive function compared with the IL-33(−) patients (1-year ΔMMSE of IL-33(+) vs IL-33(−), 0.16 ± 1.6 vs − 1.5 ± 2.6, *P* = 0.006). We then examined whether the IL-33(+) patients had lower levels of Aβ or tau that may contribute to their cognitive preservation. Figure 2 shows that the IL-33(+) patients did not have lower levels of Aβ_1-42_, Aβ_1-40_, t-Tau, or p-Tau. Instead, the IL-33(+) patients had higher levels of Aβ_1-40_ than the IL-33(−) patients (53.5 ± 4.3 vs 51.3 ± 4.1, *P* = 0.035). When analyzing Aβ_1-42_/Aβ_1-40_ ratio, group difference did not reach significance (IL-33(+) vs IL-33(−), 0.33 ± 0.04 vs 0.34 ± 0.03, *P* = 0.130). We also examined whether the IL-33(+) patients had lower expression of ApoE ε4. The proportion of ApoE ε4 expression did not differ significantly between groups [IL-33(+) vs IL-33(−), 15.4% vs 22.9%, *P* = 0.442].

**Fig. 3:**
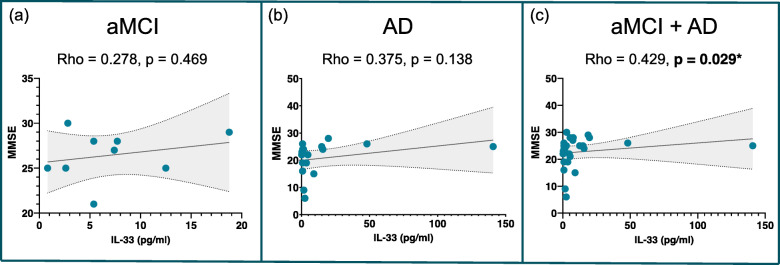
The positive correlation between the levels of IL-33 and MMSE in the patient group with 95% confidence intervals. Abbreviations: *aMCI* amnestic mild cognitive impairment, *AD* Alzheimer’s disease, *IL* interleukin

Figure 3 illustrates the Spearman correlation analysis for the association between the follow-up MMSE and the IL-33 levels in the patient group. A significant positive correlation was observed in the patient group (aMCI + AD) (rho = 0.429, *P* = 0.029). The patients with aMCI and the patients with AD also showed positive correlations, but they were not statistically significant.

We next assessed the levels of IL-1β and IL-1RA between IL-33(+) and IL-33(−) patients. We found that the IL-33(+) patients had higher levels of IL-1β than IL-33(−) patients (0.15 ± 0.14 vs 0.10 ± 0.10, *P* = 0.011). The levels of IL-1RA was also higher in IL-33(+) patients than in IL-33(−) patients but without statistical significance (96.07 ± 40.24 vs 95.64 ± 94.02, *P* = 0.189).

### The association between cognitive preservation and Aβ, tau, and ApoE ε4

**Fig. 4:**
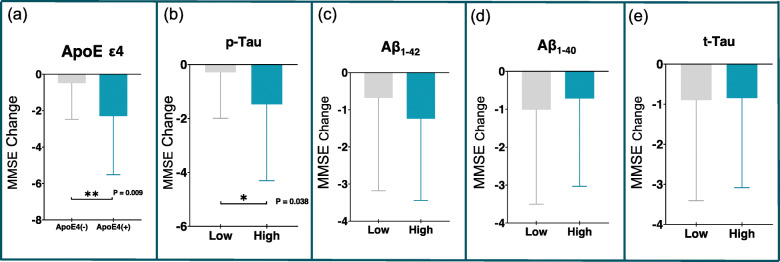
Cognitive preservation was not associated with ApoE4, p-Tau, Aβ_1-42_, Aβ_1-40_, and t-Tau, while ApoE ε4 expression and high levels of p-Tau had significantly cognitive decline than their comparators. **a** MMSE change between ApoE4 expression vs ApoE4 non-expression. **b** MMSE change between high levels of p-Tau vs low levels. **c** MMSE change between high levels of Aβ1-42 vs low levels. **d** MMSE change between high levels of Aβ1-40 vs low levels. **e** MMSE change between high levels of t-Tau vs low levels. Error bars indicate standard deviation. Abbreviations: *Aβ* amyloid β, *MMSE* Mini Mental State Examination, *p-Tau* phosphorylated Tau 181, *t-Tau* total Tau

Aβ, tau, and ApoE ε4 are well-known risk factors of AD, and thereby, we sought to examine whether cognitive preservation was associated with ApoE ε4 non-expression or lower levels of Aβ and tau. The patient group was divided into two groups: ApoE ε4(+) and ApoE ε4(−). Additionally, the patient group was divided into two categories—high and low—according to the calculated mean value of f Aβ_1-42_, Aβ_1-40_, t-Tau, and p-Tau. Figure 4 shows that ApoE ε4 expression (*P* = 0.009) and higher levels of p-Tau (*P* = 0.038) were significantly associated with cognitive decline compared with ApoE ε4 non-expression and lower levels of p-Tau, respectively. ApoE ε4 non-expression and lower levels of Aβ_1-42_, Aβ_1-40_, t-Tau, and p-Tau were not associated with cognitive preservation.

### IL-33(−) between aMCI with AD conversion vs aMCI without AD conversion

Finally, we sought to test whether IL-33(−) patients may have higher risk of AD conversion. The aMCI patients without any decline between the first and second MMSE were defined as non-AD converter, while the AD patients with first MMSE > 27 (middle school), > 23 (primary school), and > 20 (illiteracy) were defined as AD converters. For example, an AD patient with 9-year education level had first MMSE score of 27 and second MMSE score of 24 which were allocated into AD converter. Thus, we identified 11 AD converters and 15 non-AD converters. We next examined the proportion of IL-33(−) between AD converters and non-AD converters. Our results showed that AD converters had higher proportion of IL-33(−) than non-AD converters (90.9% vs 53.3%, *P* = 0.04).

## Discussion

The CNS has the highest levels of IL-33 expression in all human organs [15–17], and recent basic and preclinical studies have reported its extended physiological and pathophysiological role in CNS development [17, 19], recovery [16, 28, 29], and disease [15, 18, 21]. Here, we further our understanding of the IL-33 in human AD research. The main findings of this study were as follows: (1) most of healthy controls did not have detectable levels of peripheral IL-33; (2) the IL-33 expression showed positive linear trend between healthy controls, aMCI, and AD; (3) the IL-33-expressing patients preserved their cognitive function over 1-year period; (4) the cognitive preservation was not associated with the levels of Aβ and tau and the expression of ApoE ε4; and (5) the aMCI patients with subsequent AD conversion had higher proportion of IL-33 non-expression.

In our study, the aMCI and AD patients had higher peripheral levels of Aβ_1-42_, t-Tau, and p-Tau than controls, indirectly reflecting their central neurodegenerative conditions. The aMCI and AD patients lacking IL-33 expression revealed significantly cognitive decline, while the patients with IL-33 expression preserved their cognitive function over 1-year period. This finding was consistent with the bidirectional relationship between IL-33 deficiency and neurodegeneration in several studies, including the following: (1) mice lacking IL-33 had persistent inflammation and severe neurodegeneration in retinal detachment [30]; (2) IL-33 deficiency mice failed to repair deoxyribonucleic acid damage of aged neuron, resulting in neurodegeneration and tau abnormality [19]; (3) mice lacking IL-33 were found to have impaired recovery after CNS injury [16]; and (4) IL-33 treatment rescued contextual memory deficits in AD mouse models [18]. Collectively, our study provided the first human evidence that linking IL-33 to neurodegeneration in the aMCI and AD patients.

Comparisons between our study and the animal studies with manipulation of IL-33 revealed inconsistent findings. An animal study showed that peripheral administration of IL-33 could reduce soluble Aβ levels and reverse cognitive decline in AD mouse models [18]. Our study design was observational in nature, and we could not observe the effects of exogenous IL-33 administration in rescuing AD mice-related brain neuropathology [18]. Our aMCI and AD patients with IL-33 expression did not have lower levels of Aβ and tau. However, we found that the IL-33-expressing patients had higher levels of IL-1β. IL-33 and IL-1β belong to the IL-1 family, and IL-1β has been shown to reduce amyloid plaque pathology in AD mouse models [31, 32]. Moreover, we found a significant and positive association between IL-33 expression and cognitive preservation, and the levels of IL-33 were positively associated with the MMSE scores. These findings indirectly support the role of IL-33 in cognitive preservation in patients with aMCI or AD. Future longitudinal studies are needed to warrant the link between Aβ and IL-33 in human studies.

Our data with follow-up were consistent with a human study addressing the baseline differences between MCI with subsequent AD conversion and MCI without subsequent AD conversion [20]. Compared with AD converters, AD non-converters had increased baseline levels of IL-33^+^ cell that was also positively correlated with baseline bilateral hippocampus volumes [20]. These findings indirectly supported the association between IL-33 deficiency and neurodegeneration. Indeed, in our study, the AD converters had higher proportion of IL-33 non-expression than the AD non-converters.

Although our study found a positive linear trend of IL-33 expression among heathy controls (3.8%), aMCI (25.7%), and AD (43.6%), the longitudinal changes of IL-33 expression remain unclear in aMCI and AD. Several lines of evidence suggest that AD-related neurodegeneration begins 20 years or more before the affected individual experiences noticeable symptoms [1]. The IL-33 non-expression in aMCI and AD may be a condition of deficiency (insufficient production) or a consequence of depletion (excessive consumption). A previous study found lower baseline levels of IL-33^+^ cell in MCI patients with AD conversion [20], which was consistent with our study showing a higher peripheral IL-33 non-expression in aMCI patients with AD conversion than non-AD converters. Another human genetic study also reported lower levels of IL-33 expression in the brain of AD cases than controls [21]. Taking these findings together, insufficient production of IL-33, rather than IL-33 depletion, might be associated with the risk of AD conversion and rapid cognitive decline. However, future studies need to prospectively examine the levels of IL-33 in the preclinical stage of aMCI and AD.

### Limitations

First, the bioactivity of IL-33 is limited in blood [33]. A study measured the serum levels of IL-33 in 30 healthy controls and found that all of the samples were undetectable (lowest limit of detection, 75 pg/mL) [33]. Future study addressing the role of IL-33 in AD can simultaneously measure IL-33 and its receptor ST2. Second, we used a multiplex bead-based assay method to measure the plasma levels of IL-33; however, this method does not specify which IL-33 isoforms (full-length or cleaved) were measured. Several studies have reported that the size of the IL-33 molecules strongly influences their bioactivity [34–36]. For example, the mature forms of IL-33 (cleaved) are 30-fold more potent than full-length IL-33 for activation of innate lymphoid cells [35]. Therefore, future studies should consider a Western blot analysis to identify different forms of IL-33 in association with aMCI and AD. Third, our sample size was small; therefore, our findings need to be validated by future large-scale studies. Fourth, several biomarkers were only measured once. Future studies should assess the longitudinal changes of IL-33, Aβ, and tau in association with the cognitive decline. Finally, the correlation between the CSF levels of IL-33 and the peripheral levels of IL-33 remains to be determined.

## Conclusions

This is an early foray into the association of IL-33 in human AD research, indicating an association between IL-33 expression and cognitive preservation in aMCI and AD patients. Unanswered key questions include the underlying mechanism of IL-33 deficiency in mediating cognition decline in aMCI and AD and the trajectory of IL-33 expression from preclinical AD stage to full-blown AD. In conclusion, our findings suggest that IL-33 or its associated signaling pathways may represent a new treatment paradigm for AD.

## Data Availability

The datasets generated and/or analyzed during the current study are available from the corresponding author on reasonable request.
